# A Metabolomics Approach Reveals Immunomodulatory Effects of Proteinaceous Molecules Derived From Gut Bacteria Over Human Peripheral Blood Mononuclear Cells

**DOI:** 10.3389/fmicb.2018.02701

**Published:** 2018-11-13

**Authors:** Noelia Cambeiro-Pérez, Claudio Hidalgo-Cantabrana, Marco A. Moro-García, Rebeca Alonso-Arias, Jesús Simal-Gándara, Borja Sánchez, Elena Martínez-Carballo

**Affiliations:** ^1^Nutrition and Bromatology Group, Department of Analytical and Food Chemistry, Faculty of Food Science, University of Vigo, Ourense, Spain; ^2^Department of Microbiology and Biochemistry of Dairy Products, Instituto de Productos Lácteos de Asturias, Consejo Superior de Investigaciones Científicas, Villaviciosa, Spain; ^3^Department of Immunology, Hospital Universitario Central de Asturias, Oviedo, Spain

**Keywords:** bacterial peptides, human peripheral blood mononuclear cells (PBMCs), untargeted metabolomics, LC-ESI-QTOF-MS, host immunomodulation

## Abstract

There are strong evidences that probiotics influence the immune status of the host, in a strain-specific manner, acting in the gastrointestinal tract. On the hypothesis that certain extracellular proteins and peptides from gut bacteria may mediate part of this immunomodulation and assuming they are able to diffuse through the mucus layer and interact with immune cells we have developed this work. Our study attempts to understand the immunomodulatory mechanisms of (i) Pext, the extracellular protein fraction of *Lactobacillus acidophilus* DSM20079^T^, (ii) HM14, a peptide encrypted in an extracellular glycoside hydrolase from *Bifidobacterium longum* NCIMB 8809 and (iii) *Escherichia coli* O111:B4 lipopolysaccharide (LPS), a well-known pro-inflammatory molecule, over human peripheral blood mononuclear cells (PBMCs). An untargeted LC-ESI-QTOF-MS metabolomics approach was applied to reveal intracellular changes in treated-PBMCs isolated from healthy donors. Differences in NADH arrest, NAD^+^ concentration reduction, as well as increases in palmitic acid and methanephrin were observed in HM14 and Pext treated-cells compared to those stimulated with LPS. This would support an anti-inflammatory molecular mechanism of action of such proteinaceous molecules. Moreover, this methodology has confirms the importance of metabolomics approaches to better understanding immune cell responses to gut bacterial-derived molecules.

## Introduction

According to the Food and Agricultural Organization of the United Nations, probiotics are defined as “live microorganisms, which when administered in adequate amounts confer health benefits on the host” (FAO/WHO, 2002). Among the different traits that can be held by a probiotic strain, immunomodulation is widely reported (Holmes et al., 2012; Hemarajata and Versalovic, 2013; Quévrain et al., 2015) but at the same time is one of the most rare and strain-specific beneficial effects over host health (Hill et al., 2014). This immunomodulation mechanism, performed in the gastrointestinal tract, could be achieved through changes in cytokine production and modulation of signaling pathways in intestinal epithelial and immune cells (Hemarajata and Versalovic, 2013).

During the last years, we have worked on the hypothesis that certain extracellular proteins, and peptides derived from by the action of intestinal proteases, may mediate some of the immunomodulatory mechanisms of probiotic bacteria, as these molecules are able to diffuse through the mucus layer and interact with immune cells, notably in the last sections of the small intestine (Sanchez et al., 2010). The existence of an influence of probiotic intake over the immune status of the host is well-known, and as an example soluble factors from *Lactobacillus reuteri* inhibit production of pro-inflammatory cytokines and signaling immune cells Thomas et al. (2012). S-layer protein A (SlpA) produced by the probiotic *Lactobacillus acidophilus* NCFM strain is known to bind the surface lectin DC-SIGN, which is expressed on the surface of intestinal dendritic cells leading to an increase in the production of the anti-inflammatory cytokine IL-10 and an inability to induce further T-cell proliferation (Konstantinov et al., 2008). In this work, we have used the extracellular protein fraction of *L. acidophilus* DSM20079^T^ (Pext). This fraction is characterized by containing a major protein, SlpA, which accounts for more than the 95% of the total protein production (Sánchez et al., 2011). SlpA abundance makes Pext a very suitable fraction for the study of the molecular mechanism of action of extracellular proteins. In addition, the proteolytic activity of this bacterium avoids interference of other proteins present in the bacteria growth media, some of them possessing also immunomodulatory properties such as porcine serpin (Urdaci and Sánchez, 2009).

Our own work has identified encrypted peptides which, when released by the action of intestinal proteases, interact with dendritic cells rendering an anti-inflammatory profile and modulating functional properties such as the capacity to imprint different home markers on the surface of T-cells (Bernardo et al., 2012). With this hypothesis in mind, we have generated a bioinformatics resource denominated MAHMI (Molecular mechanism of Action of the Human Microbiome) database (http://www.mahmi.org), which contains all the peptide generated by the *in-silico* action of intestinal proteases over the non-redundant protein catalog originated from the METAHIT project (Blanco-Míguez et al., 2017). This resource was queried against a knowledge database of bioactive proteins, allowing us to identify potential immunomodulatory peptides encrypted in larger proteins. This was the case of FR-16 and LR-17, 16-mer and 17-mer peptides derived from *Bifidobacterium longum* DJ010A and *Bacteroides fragilis* YCH46 respectively, which polarized human peripheral blood mononuclear cell (PBMCs) responses toward increases in the Th17/Th1 balance (Hidalgo-Cantabrana et al., 2017). The same resource allowed us to identify peptide HM14, encrypted in an extracellular glycoside hydrolase from *B. longum* and which was predicted as a potential anti-inflammatory peptide (unpublished results).

Metabolomics is an omics technique, which aim is the comprehensive analysis of the biological system's metabolome, identifying the wide variety of low molecular weight metabolites that are chemically transformed during metabolism (Patti et al., 2012). Moreover, metabolomics cell phenotyping, the identification of metabolic events, and the interpretation of cell responses to different signals is the correct approach of endo-metabolome (León et al., 2013). Untargeted metabolomic strategy is intended to detect as many known and unknown metabolites as possible in a biological sample through magnetic resonance (NMR) or mass spectrometry (MS) platforms (Shah et al., 2012). This approach provides the opportunity to observe changes when biological samples or clinical states are compared.

In this context, the present study attempts to evaluate changes in the intracellular metabolite composition of PBMCs exposed to different bacterial components by using a LC-EI-QTOF-MS methodology and an untargeted profiling approach. PBMCs exposed to lipopolysaccharide (LPS) from *Escherichia coli* O111:B4, a well-known pro-inflammatory molecule, and two probiotic-derived molecules: a peptide derived from an extracellular glycoside hydrolase from *B. longum* NCIMB 8809 (HM14) and the extracellular protein fraction from *L. acidophilus* DSM20079^T^ (Pext), were submitted to this untargeted metabolomics approach to infer changes in the metabolic pathways. Main results are presented next.

## Experimental

### Chemicals, solutions and materials

Standards of DL-Norvaline, Succinic acid-2,2,3,3-d_4_ and trans-Cinnamic-d_7_ acid, used as surrogates, were purchased from Sigma Aldrich (Missouri, United States). Each standard was prepared at 100 mM in water or methanol depending on the solubility of the chemical. Surrogate solution and a mix of standards were prepared in methanol at 1.0 mM. These solutions were stored in amber flasks at −20°C. RPMI 1640 medium containing 2.0 × 10^−3^ M l-glutamine and HEPES (Lonza, Basilea, Switzerland) was supplemented with 10% fetal bovine serum (FBS) (ICN Flow; Costa Mesa, CA, United States) and antibiotics. Phosphate buffered saline (PBS) was purchased from Oxoid Limited (Hampshire, UK) and Ficoll-Paque Plus (Lymphoprep) from Nycomed (Oslo, Norway). Flow cytometry reagents: anti-CD4 (PerCP), anti-CD8 (PE), anti-CD19 (APC), anti-CD56 anti-CD16 (FITC) antibodies and 7-aminoactinomycin D (7-AAD) were purchased from eBioscience (San Diego, CA, USA). The bacterial peptide HM14 from *B. longum* NCIMB 8809 was synthesized at GeneCust facilities to >95% purity (Ellange, Luxemburg). The extracellular protein fraction from *L. acidophilus* DSM20079^T^, denominated Pext, was isolated from spent growth supernatant as described previously (Sánchez et al., 2009). Lipopolysaccharides (LPS) from *Escherichia coli* O111:B4 purified by phenol extraction was purchased from Sigma-Aldrich (Madrid, Spain).

Analytical grade C-45 nitrogen and helium was supplied by Carburos Metálicos (Vigo, Spain). Additional equipment included a TurboVap (LifeScience, Hopkinton, MA, United States), an ultrasonic bath (P-Selecta, Barcelona, Spain), an analytical precision scale (Sartorius, Madrid, Spain) and a vortex shaker (Heidolph, Barcelona, Spain). Disposables used were micropipettes (200–1000 μL) and injection vials (2.0 mL) furnished with screw caps and PTFE-lined butyl rubber septa and inserts (0.35 mL). The used solvents were high quality suitable to the chromatographic analysis: Acetone (CHROMASOLV for HPLC ≥99.8%), Methanol (CHROMASOLV for HPLC ≥99.9%), Chloroform (CHROMASOLV for HPLC ≥99.9%), Water (CHROMASOLV for HPLC ≥99.9%), Acetonitrile (CHROMASOLV for HPLC ≥99.9%).

Ethics approval for this study (reference code AGL2013-44039-R) was obtained from the Regional Ethics Committee for Clinical Research (*Comité de Ética de la Investigación del Principado de Asturias*) in compliance with the Declaration of Helsinki. Samples used in this study were obtained from anonymous donors from our blood donation system.

### Peripheral blood mononuclear cells isolation

To analyse the metabolomics pattern induced by bacterial components in the human immune system, PBMCs were isolated from the buffy coat of 5 healthy donors obtained from the Community Center for Blood and Tissues of Asturias (Oviedo, Spain). In short, 5.0 mL of buffy were diluted with equal volume of PBS added on top of 5.0 mL Ficoll-Hypaque Plus for gradient separation. Cells were separated by centrifugation (1800 rpm, 30 min) with two further steps of washing with PBS; the first one at 1200 rpm, 10 min (to discard platelets) and second one at 1500 rpm, 5.0 min. Then, PBMCs were counted in Neubauer chamber and adjusted to 2.5 × 10^7^ mL^−1^ in RPMI 1640 supplemented with 10 % FBS and antibiotics.

### Co-cultivation of peptides and PBMCs

The PBMCs were cultivated in flat bottom 12-wells microplates using 2.0 mL cell suspension at high density (2.5 × 10^7^ cells mL^−1^ per well). Positive LPS (1.0 μg mL^−1^) and negative controls (no stimulus) were included for each donor. The peptide HM14 was added at a final concentration of 50 μg mL^−1^ (Hidalgo-Cantabrana et al., 2017). The Pext fraction from *L. acidophilus* DSM20079^T^ was added at a final concentration of 10 μg mL^−1^, as the concentration giving the highest responses in our immunomodulatory tests (unpublished results). In addition, blank samples (with no cells) were included in the study (*N* = 5). The microplates were incubated for 2 days at 37°C with 5.0 % CO_2_.

### Sample preparation

#### Quenching extraction

**Figure 1:**
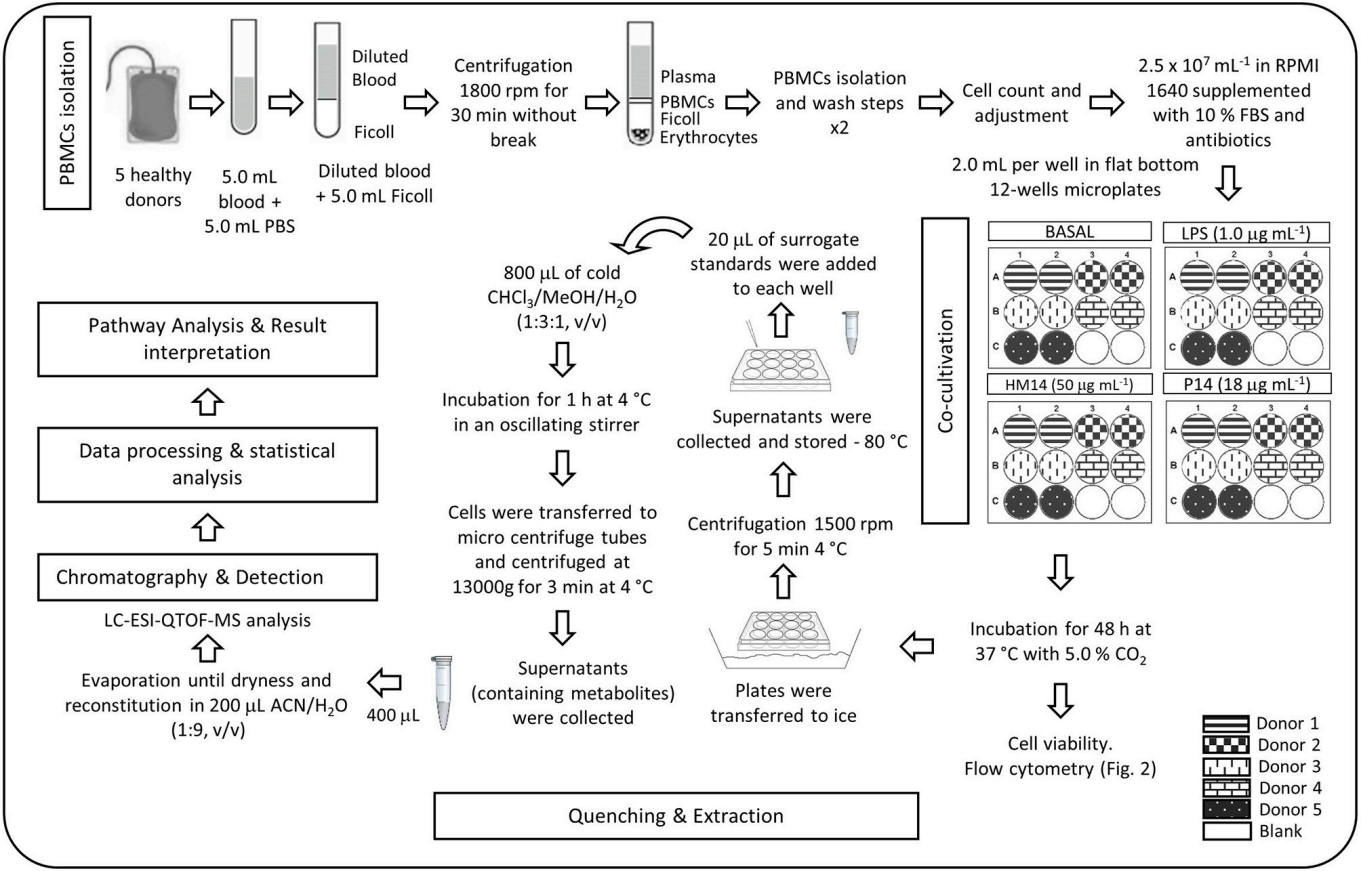
Diagram flow of the experimental protocol.

After 48 h culture of PBMCs cells were submitted to a quenching protocol to preserve metabolite levels of cells to avoid degradation. The importance of quenching metabolic processes during cell harvesting is widely reported in the literature (Dettmer et al., 2011; McNamara et al., 2012; León et al., 2013). Shortly, cells were transferred to ice and harvested (1500 rpm, 5.0 min, 4.0 °C). Supernatants were collected and stored at −80°C for further analysis. Cells were then resuspended for 5.0 min in 20 μL of cold surrogate standards solution. Then, 800 μL of a pre-cooled extraction solvent chloroform/methanol/water (1:3:1) was added, and cell suspension was incubated for 1.0 h, at 4.0°C in an oscillating stirrer. Subsequently, cells were transferred to cold micro centrifuge tubes and supernatants (containing intracellular metabolites) were harvested (13,000 g, 3.0 min, 4.0°C). Prior to injection, 400 μL of supernatants were reduced to dryness under gentle nitrogen stream, redissolved in 200 μL ACN:H_2_O (1:9, v/v) and, finally, transferred to vials for LC-QTOF-MS analysis. Figure 1 summarizes a diagram flow of the selected experimental protocol.

#### Quality control sample

Two different types of Quality Control (QC) samples have been employed (QC and QC surrogate). QC sample was made by pooling 45 μL from each of analyzed samples and surrogate QC sample (QC surrogate), by spiking with surrogate standards to the QC sample.

### Flow cytometry analysis

Lymphocyte subsets from PBMCs were analyzed in a BD Accuri C6 flow cytometer (BD Biosciences, Allschwil, Switzerland) at starting point (0 h) and after 48 h incubation. Cells were labeled with human anti-CD4 (PerCP) antibody to target CD4^+^ T-lymphocytes, anti-CD8 (PE) for CD8^+^ T-lymphocytes, anti-CD19 (APC) for B-lymphocytes, anti-CD56 and anti-CD16 (FITC) for Natural killer (NK) cells. Also, after 48 h incubation, cells were stained 7-AAD to checked cell viability, as 7-AAD labeled DNA only if the cells are not alive. Briefly, 1.0 × 10^6^ cells were collected into a flow cytometry tube and washed with PBS (1500 rpm, 5.0 min). Cell pellet was stained with human antibodies and 7-AAD for 20 min at 4.0°C. Then, cells were washed with 1.0 mL of PBS and resuspended in 200 μL of PBS for flow cytometry quantification.

### Chromatography and detection technique

For the untargeted screening, an Agilent (Wilmington, DE) 1200 Series LC was used. The LC system was interfaced to a quadrupole-time-of-flight (QTOF) HRMS model Agilent 6520 equipped with a Dual electrospray (ESI) ion source, operating in positive ion mode. Nitrogen was used as nebulizing (42 psig) and drying gas (350°C, 5.0 L min^−1^) in the dual ESI source. The QTOF instrument was operated in extended dynamic range 2.0 GHz mode. This mode provides a full width at half-maximum (fwhm) resolution of ca. 10600 at m/z 118.0862 and ca. 16900 at m/z 922.0097. A reference calibration solution, supplied by Agilent, was continuously sprayed in the source during the chromatographic run, providing the required accuracy of mass assignations. Chromatographic separations were performed with a Kinetex™ 2.6 μm C18 (100 Å, LC Column 100 × 2.1 mm) and with a Kinetex™ 2.6 μm Polar C18 (100 Å, LC Column 100 × 2.1 mm; Phenomenex, Torrance, CA, United States). The optimal working conditions were obtained with the Polar C18, in which the solvents consisted of A: 0.10% HCOOH in H_2_O and B: 0.10% HCOOH in acetonitrile. The gradient was: 100% A during 2.0 min, change to 100% B in 18 min and hold during 5.0 min, change to initial conditions in 0.10 min and hold for 10 min giving an analysis time of 35 min at 35°C. The injection volume was set to 5.0 μL by LC flow rate of 0.20 mL min^−1^. Adjustment and operation of the instrument were performed by means of MassHunter Workstation Software B.07.00.

### Data processing and statistical analysis

The resulting raw data files (.d files) were processed using MassHunter Profinder Software B.08.00 (Agilent Technologies) with Batch Recursive Feature Extraction (BRFE) algorithm. This option includes two steps: firstly, performs chromatographic deconvolution of samples to find features (compounds) through an untargeted Molecular Feature Extraction (MFE) algorithm. It finds co-eluting ions that are related such as isotopes, common adducts ([M+H]^+^, [M+Na]^+^, [M+K]^+^) and dimmers, besides filtering noise and create a compound chromatogram for the group of ions, unifying all these ion signals into one value, so that one feature is equivalent to one compound. Followed by mass and retention time alignment of molecular features across all of the selected samples. Last, Find by Ion feature extraction (FbI) algorithm uses the median values derived from the MFE step to perform a targeted extraction to improve the reliability in finding features. In this way, it improves the accuracy of the feature extraction by reducing the number of false negatives and false positives in the dataset. The MFE parameters included for feature extraction a minimum peak height of 150 counts, a tolerance window for compound binning and alignment of 0.50 min for retention time and a mass accuracy of 20 ppm for m/z values. FbI parameters for peak filtering were set a minimum absolute height at 1,000 counts.

Extracted compounds, comprised of a neutral mass, retention time, and abundance, were exported as compound exchange files (.CEF) for further feature alignment, data processing and multivariate statistical analysis using Mass Profiler Professional B.14.08 (Agilent Technologies). Data were filtered with a retention time tolerance of 0.15 min and mass tolerance of 2.0 mDa. One way ANOVA and Tukey's honest significance difference (HSD) *post hoc* test were applied to identify which entities were responsible for significant differences between conditions (*p*-value < 0.001). Compounds differing by a fold change >2 between groups were considered for putative identification.

Multivariate statistical analysis was also performed for comparative metabolite profiling. Unsupervised methods as Principal component analysis (PCA) and hierarchical cluster analysis (HCA) were used for data exploration and visualization, providing information about the presence of outliers, sample dispersion and data clustering. Samples were classified into discrete classes also by supervised Partial Least Square Discriminant Analysis (PLS-DA).

### Metabolite identification and pathway analysis interpretation

Statistically significant metabolites selected by MPP were putatively identified through MassHunter METLIN Metabolite PCDL (Agilent Technologies). In case of unknown metabolites, molecular formulas were generated by the software. Mass accuracy tolerance of 5.0 ppm was used as mass window for database search. Also, other freely available online databases were employed as the Human Metabolome database (HMDB; http://hmdb.ca/), METLIN database (http://metlin.scripps.edu/) and KEGG database (http://www.genome.jp/kegg).

Biological interpretation was performed through the Pathway analysis tool of MPP software that allows searching the annotated metabolites in the curated database of human metabolic pathways BioCyc (http://www.biocyc.org/).

## Results and discussion

### Lymphocyte quantification and cell viability

**Figure 2:**
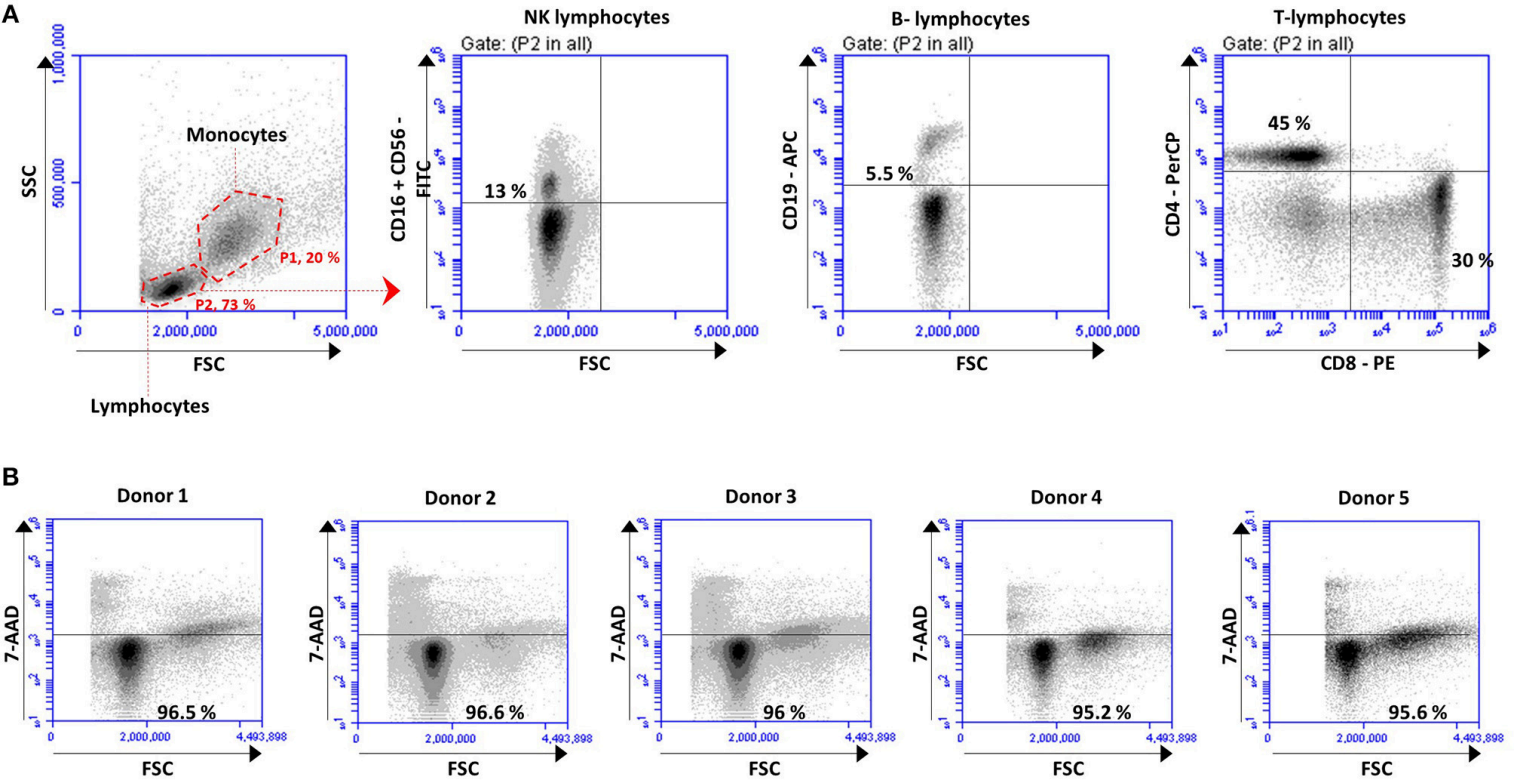
PBMCs isolated from the buffy of 5 healthy donors were analyzed before and after co-culture for 48 h to **(A)** quantified lymphocyte subsets and **(B)** cell viability. **(A)** Gating strategy used to identified monocytes (P1, 20%), lymphocytes (P2, 73%) and to quantified the different lymphocytes subsets: NK lymphocytes (CD16^+^ CD56^+^), B-lymphocytes (CD19^+^), CD4 T-lymphocytes (CD4^+^) and CD8 T-lymphocytes (CD8^+^). Density plots correspond to one representative donor. The percentage of positive cells in each subset is expressed based on the gate of lymphocytes (P2). **(B)** Cell viability analyses performed after 48 h based on the percentage of cells of each donor not labeled with 7-AAD.

Total lymphocytes and lymphocytes subpopulations were quantified at the initial point of the experiments and after 48 h incubation, as depicted for one representative donor in Figure 2A. Results showed the percentage of positive cells for each subset population. As expected, total lymphocytes (Figure 2A, first plot) were similar between the five healthy donors at initial point (0.0 h) (75.78% ± 4.31) and remained stable after 48 h incubation (72.53% ± 10.85). The different lymphocytes subsets, quantified with human antibodies labeling, remained stable after 48 h incubation in each case of study. The average (mean ± SD) of each lymphocytes subset referred to the lymphocytes gate was 13% ± 3,85 NK cells, 5.5% ± 1.43 B-lymphocytes, 45% ± 8.88 CD4 + T cells, 30.10% ± 7.07 CD8 + T cells, as depicted for one representative in Figure 2A.

Cell viability was measured by flow cytometry based on 7-AAD labeling (Figure 2B). The cell viability, quantified as the percentage of cells not labeled with 7-AAD, remained very high (96.08% ± 0.64) after 48 h incubation with no differences between the five donors (Figure 2B).

All in common established that lymphocytes subsets distribution and cell viability were not affected by high the number of cells assayed in each well, neither by the potential effect of the HM14 bacterial peptide from *B. longum*, Pext extracellular protein from *L. acidophilus* or LPS, at least during the incubation time tested. So on, it is expected that PBMCs metabolism remain closed to natural conditions, as closed as *in vitro* test support, and differences in the metabolism pattern will be directly related with the immune modulation capability of the studied stimuli.

### Non-targeted analytical method

The seven parameters established by the international guidelines for analytical method validation such as accuracy, precision, specificity, limit of detection and quantification, linearity and range (US FDA, 2000, 2015; Thompson et al., 2002; ICH Q2A, 2005) are not adjusted to the aim of the non-targeted metabolomic approach. For this reason, researchers have proposed various strategies to validate the analytical methodology for untargeted metabolomics (Naz et al., 2014) and they are as follows: to validate the method in terms of accuracy and precision for selected compounds or spiked standards, the use of pooled QCs and analytical blanks during method performance. It is of great importance the employment of QC samples in metabolic profiling analysis, as well-described in the literature (Sangster et al., 2006). The idea of using QC is to have an appropriate representative sample for the matrix to be analyzed in a regular intervals throughout the analytical run in order to monitor the quality of the resulting data (Gika et al., 2016). Aware of this need, the latest guideline for Bioanalytical method validation includes QCs as bioanalytical parameters for method validation (US FDA, 2018).

Therefore, taking into account all the indications described above, was performed the methodological validation of the present study.

#### Data quality assessment

Recoveries of surrogate standards were measured in all samples ranged from 68 to 120% with relative standard deviations < 20%.

QC samples were analyzed intermittently during the analytical run every 5 samples and the acquired data was used to determine the experiment precision. Accordingly, RSDs for spiked-standards QC samples (QC surrogate) were calculated and resulted in all cases < 10%. It was reported that data should exhibit RSD lower than 20% in LC-MS analysis. Besides check that no sensitivity loss across the analytical batch was observed, it is necessary to also check for retention time precision, which was evaluated through visual inspection of overlapped Total Ion Chromatograms (TICs) of both spiked QC and QC pooled replicates.

Multivariate analysis on QC samples also provides information about the quality of the assay. PCA score plot shows that QC and QC surrogate replicates form a tighter cluster than the rest of samples, reflecting the instrumental repeatability. Note that although QC and QC surrogate samples appears to be not as close as we would expect on the PCA score plot, hierarchical clustering algorithm shows a clear relationship between both groups.

Moreover, two types of blanks were used for method quality assessment. Blanks for quenching protocol assessment (BQuench samples) and analytical blanks, which contain the injection solvent. Analytical blanks allow controlling the correct method development and identifying background features. And, on the other hand, BQuench samples allow to identify the background related to the entire methodological process from quenching to injection.

#### LC-QTOF -MS method development

Different chromatographic columns (Kinetex™ 2.6 μm C18 (100 Å, LC Column 100 × 2.1 mm) and Kinetex™ 2.6 μm Polar C18 (100 Å, LC Column 100 × 2.1 mm) (both packed with core-shell materials) and mobile phases were tested in order to study the retention of both polar and nonpolar substances. Since, reverse phase liquid chromatography (RPLC) is used for the detection of nonpolar compounds, a Kinetex Polar C18 was used in order to retain mixtures of multiple polar and nonpolar compounds. Moreover, they were also able to isolate closely related compounds, such as impurities or metabolites. A generic gradient was selected in order to identify most symmetric and intense peaks with a high resolution. Mobile phases A consisted in 5.0 mM NH_4_Ac with 0.10 % HCOOH or 0.10 % HCOOH were tested, as well as, methanol or acetonitrile as mobile phases B. Since no differences were detected using methanol as modifier with NH_4_Ac to prevent secondary retention mechanism, acetonitrile and HCOOH were selected as mobile phases in order to achieve more retention with polar compounds. In this work, QC surrogates were used for such purposes. Kinetex™ 2.6 μm Polar C18 was selected as working column because it gave the best chromatographic separation with a good peak shape. Flow rate of 0.20 mL/min, column temperature of 35°C and injection volume of 5.0 μL were also optimized.

### Data pre-processing, statistical analysis and metabolite identification

After data processing by MPP, 1155 of 2976 entities among groups were significantly differentiated after applying filtration using frequency (entities that appear in the 100 % of samples in at least one condition), *p*-value < 0.001 and fold change > 2.0.

**Figure 3:**
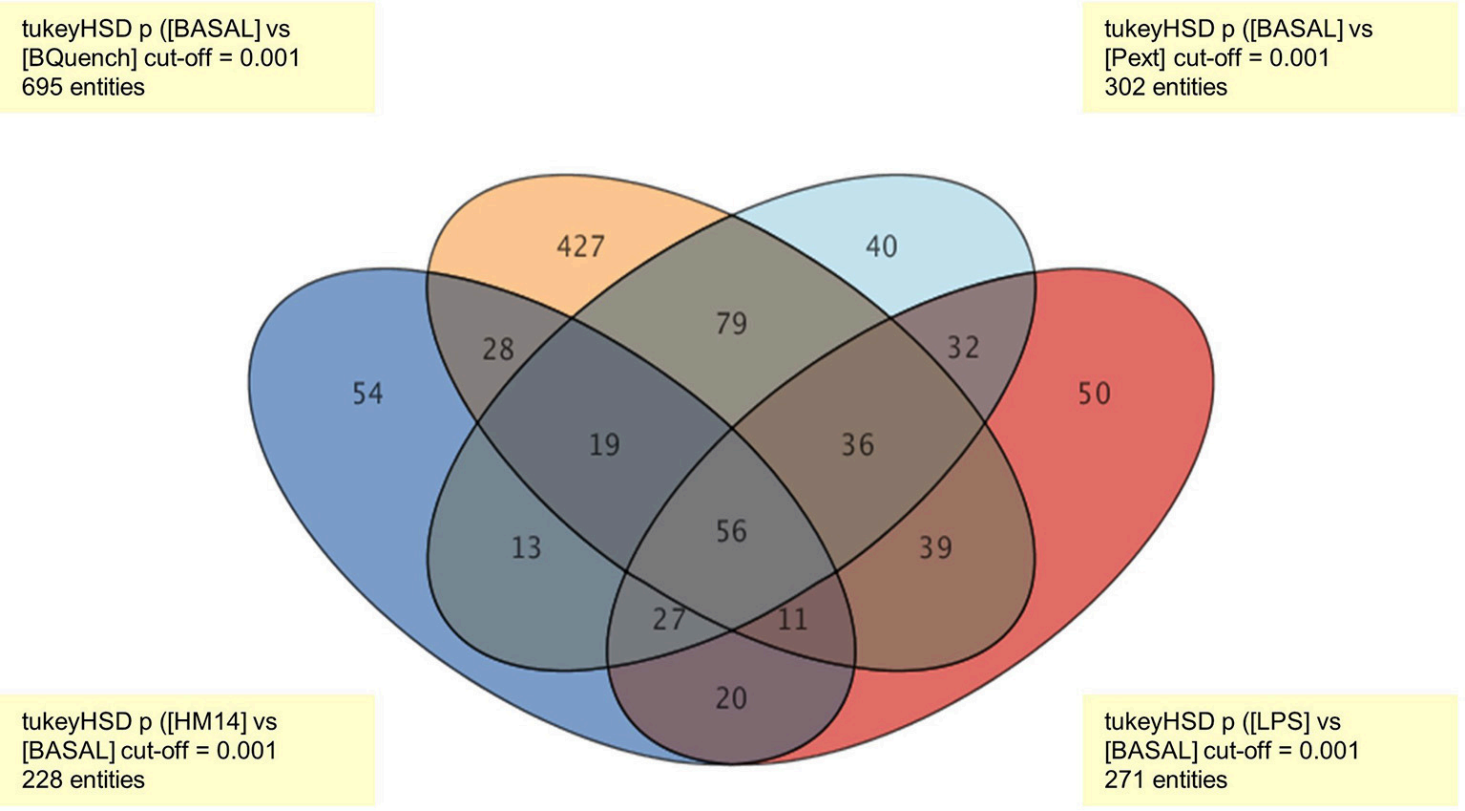
Venn diagram representing all relationships between the four groups (Pext, HM14, LPS, and Blank) in comparison to Basal conditions. The number of entities responsible for the differences between treatment groups and control were 302 for Pext, 228 for HM14 and 271 for LPS.

Tukey's honest significance difference (HSD) *post hoc* test was applied to identify which entities were responsible for significant differences in the five groups (Pext, HM14, LPS, Blank, and Basal). In order to present data in easy-to-understand visualization, a Venn diagram is given to show all possible logical relations between the four groups (Pext, HM14, LPS, and Blank) regarding to Basal condition (Figure 3). It was found that the number of entities responsible for the differences among treatment groups and control were 302 for Pext, 228 for HM14 and 271 for LPS.

**Figure 4:**
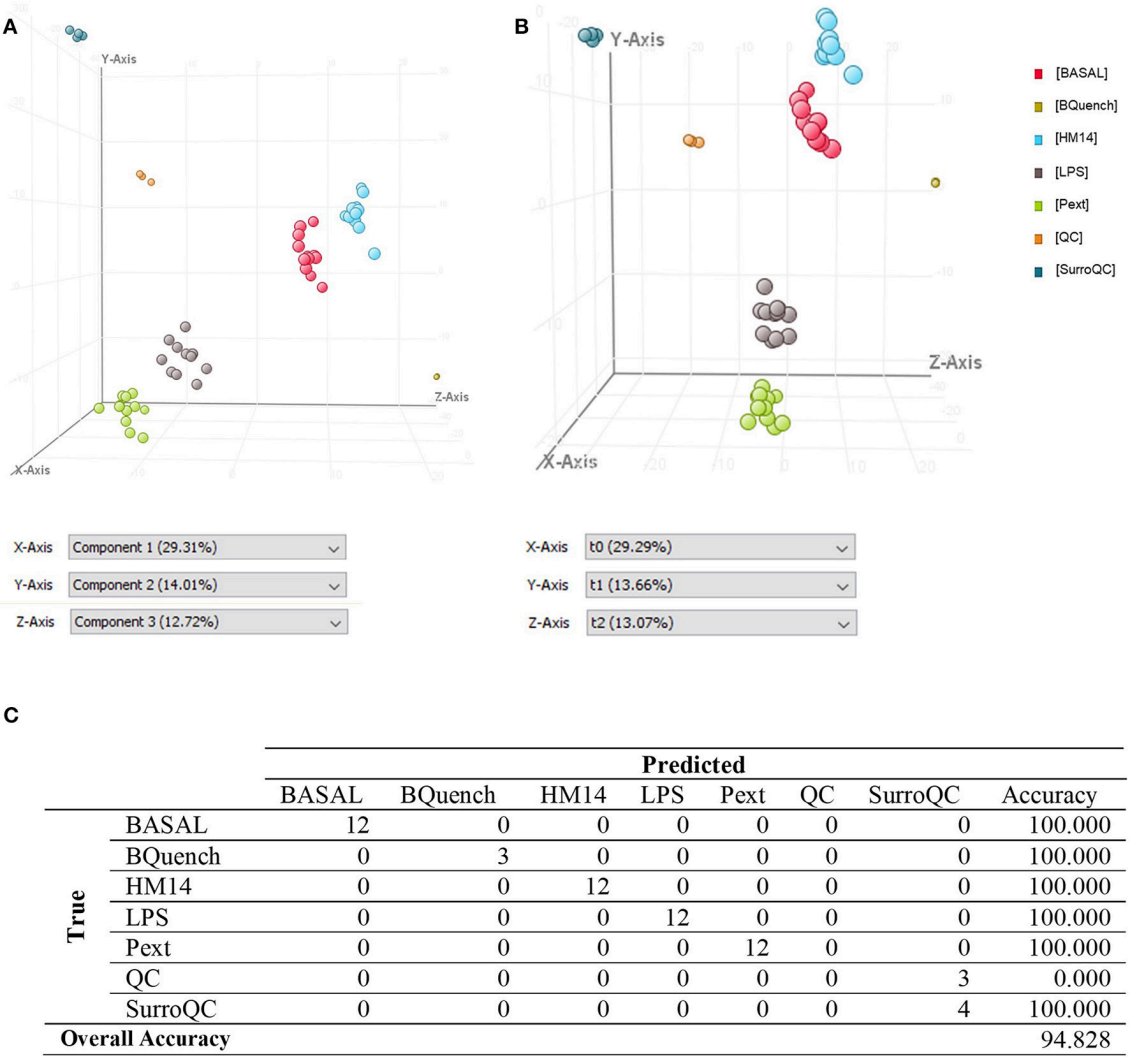
**(A)** Three-dimensional PCA score plot, **(B)** PLS-DA score plot and **(C)** Confusion matrix of the PLSDA model.

**Figure 5:**
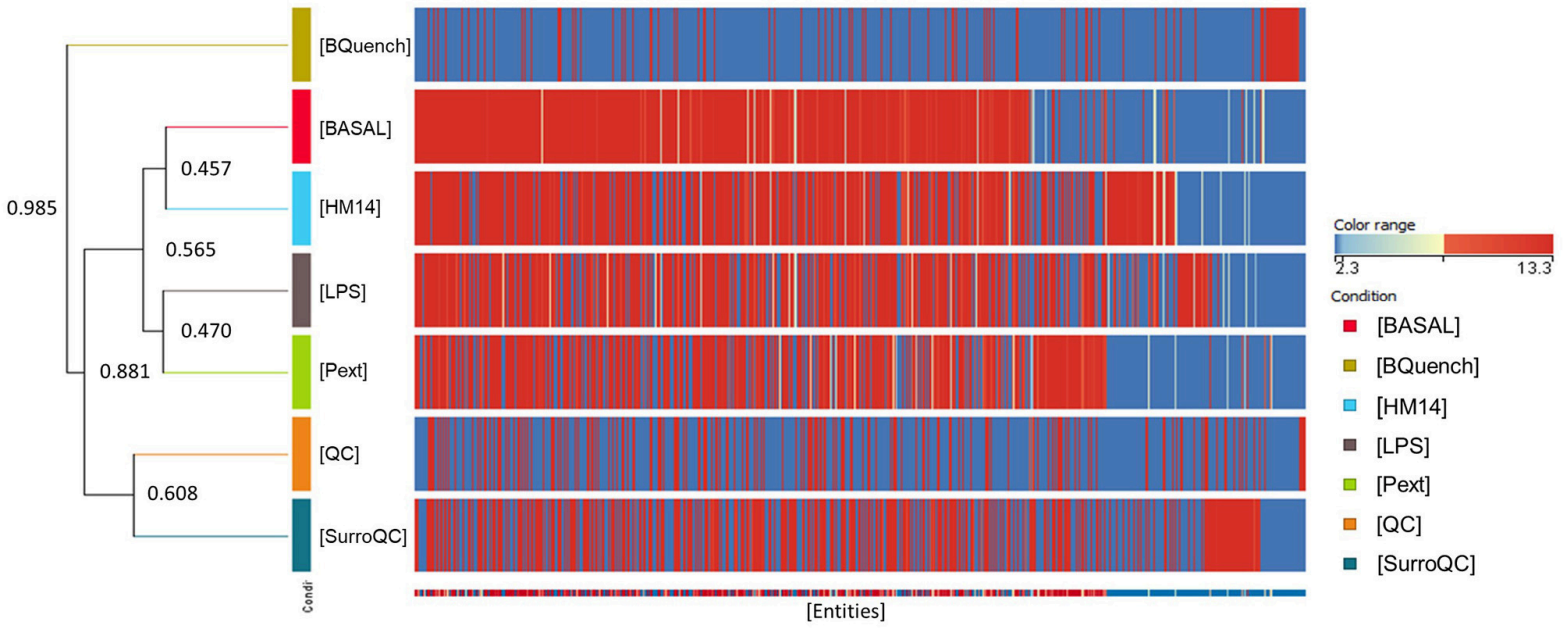
Comparison of seven groups of samples using normalized intensities of annotated metabolites. The dendogram was produced by applying a hierarchical clustering algorithm.

For multivariate analysis, about 56% of the total variance in the data was represented by the three first principal components in the PCA (Figure 4A). The 3D-PCA score plot represent each sample as a single point. No outlier samples were observed and the data showed a grouped distribution, revealing a remarkable difference between conditions. Furthermore, some lightly similarity among Basal condition and HM14 samples can be observed and both can be distinguished from LPS and Pext conditions, which seem to be also related one to each other. The cluster of QC samples provides a reliable stability and repeatability of the analysis sequence. In order to maximize the separation achieved with the PCA, a supervised analysis PLS-DA (Figure 4B) was subsequently performed. In the PLS-DA score plot, samples exhibited lower dispersion within the group, emphasizing the separation between the different clusters with an overall accuracy of the model of 94.828% (Figure 4C), *R*^2^ = 0.657 and *Q*^2^ = 0.478. Hierarchical clustering analysis was also performed by applying Pearson's Center-Absolute similarity measure and complete linkage, to produce a dendogram for clustering of sample groups using normalized intensities values of the processed data (Figure 5). Similar results to those explained in the PCA and PLS-DA are observed in the dendogram, indicated close clustering between HM14-Basal, and LPS-Pext. The dendogram represents a measure of dissimilarity with the length of the vertical lines and, therefore, provides an insight into the existing relationship among groups. Clustering the seven groups or conditions in six classes. Class I shows that BQuench group is the most dissimilar from the other groups with a dissimilarity level of 0.985. In class II is represented the dissimilarity between QC groups (SurroQC and QC) and the rest of treatment groups and control (class IV), with a dissimilarity level of 0.881, showing a reasonable difference with respect to class IV. Being the dissimilarity level among SurroQC and QC of 0.608, clustered together in class III. Most relevant conclusions were obtained from clustering observed in class IV, which explains dissimilarity between class V (clustering P14 and LPS groups with a dissimilarity level of 0.470) and class VI (clustering HM14 and Basal group with a dissimilarity level of 0.457) with 0.565.

### Pathway analysis

Pathway analysis was performed in MPP software from significantly differentiated metabolites. Those that have not been identified were excluded from this analysis by the software. Finally, a total of 50 annotated metabolites, named as compounds and classified in a total of 13 entities, were found related with different metabolic pathways included in the BioCyc database (Table 1 and Supplementary Table 1).

**Table 1:** List of 50 putatively identified metabolites after pathway analysis, which were classified in 13 entities, showing related metabolic pathways.

**Analysis**	**Pathways**	**Entity name**	**Compound name**	**FC**	**KEGG ID**	**Mass**	**RT**	**HMP ID**	**Formula**
UP -FC ([HM14] vs. [BASAL])	Triacylglycerol biosynthesis Phospholipases Sphingomyelin metabolism Choline biosynthesis III Phosphatidylcholine biosynthesis I	α phosphatidylcholine	PC(18:1(9Z)/20:0)	298820.78	C00157	797.6269	29.68	HMDB08109	C46 H91 N O8 P
			PC(15:0/18:3 (9Z,12Z,15Z))	238845.3	C00157	701.5361	29.67	HMDB07942	C41 H77 N O8 P
			PC(15:0/20:3 (8Z,11Z,14Z))	352141.22	C00157	729.5682	29.65	HMDB07948	C43 H81 N O8 P
			PC(18:0/22:5 (7Z,10Z,13Z,16Z,19Z))	7.0094786	C00157	817.6028	29.69	HMDB08056	C48 H87 N O8 P
			PC(18:0/18:1 (11Z)) Esi+29.679995	280889.97	C00157	747.597	29.68	HMDB08037	C44 H87 N O8 P
			PC(24:0/14:0)	215873.94	C00157	799.6408	29.70	HMDB08755	C46 H93 N O8 P
			PC(20:2(11Z,14Z)/ 20:1(11Z))	3.6340973	C00157	821.619	29.69	HMDB08341	C48 H91 N O8 P
			PC(22:4(7Z,10Z, 13Z,16Z)/15:0)	3.2290432	C00157	777.566	29.66	HMDB08625	C45 H83 N O8 P
			PC(20:3(8Z,11Z,14Z)/ 18:2(9Z,12Z))	2.1216183	C00157	785.5968	29.69	HMDB08402	C46 H83 N O8 P
	Triacylglycerol biosynthesis Phospholipases	α 2-lysophosphatidylcholine	LysoPC(22:5(4Z,7Z,10Z, 13Z,16Z))	4.713763	C04230	569.3519	19.33	HMDB10402	C30 H53 N O7 P
	Arginine biosynthesis IV Arginine degradation I (arginase pathway) Urea Cycle tRNA charging Glycine degradation (creatine biosynthesis) Arginine degradation VI (arginase 2 pathway) Proline Biosynthesis II (from arginine) Citrulline-nitric oxide cycle	L-arginine	Amino acid(Arg-) Esi+1.296263	3.3558748	C02385	174.1113	1.30		C6 H14 N4 O2
	Noradrenaline and Adrenaline degradation	Metanephrine	Metanephrine	2.4727805	C05588	179.0941	7.15	HMDB04063	C10 H15 N O3
	NADH Repair	(S)-NADHX	(6S)-6-Hydroxy-1,4,5,6-tetrahydronicotinamide-adenine dinucleotide	72856.64	C04856	683.1358	1.32	HMDB59644	C21 H31 N7 O15 P2
	Stearate biosynthesis I (animals)	Palmitic acid	Palmitic acid	2.0812156	C00249	238.2284	23.59	HMDB00220	C16 H32 O2
	Ceramide Biosynthesis	Sphinganine	Sphinganine Esi+18.641169	16.219389	C00836	301.2969	18.64	HMDB00269	C18 H39 N O2
DOWN -FC ([HM14] vs. [BASAL])	Phospholipases Sphingomyelin metabolism Phosphatidylcholine biosynthesis I Choline biosynthesis III	Phosphocholine	Phosphocholine	−77069.34	C00588	183.0663	1.35	HMDB01565	C5 H15 N O4 P
	Phospholipases Sphingomyelin metabolism Phosphatidylcholine biosynthesis I Choline biosynthesis III Triacylglycerol biosynthesis	α phosphatidylcholine	PC(20:2(11Z,14Z)/14:0)	−19412.555	C00157	757.5639	29.69	HMDB08328	C42 H81 N O8 P
			PC(18:2(9Z,12Z) /22:0)	−121347.46	C00157	823.6375	29.69	HMDB08150	C48 H93 N O8 P
			PC(20:1(11Z)/ 16:0)	−18.664034	C00157	787.6126	29.68	HMDB08298	C44 H87 N O8 P
			PC(18:2(9Z,12Z)/ 18:1(9Z))	−870785.8	C00157	783.5799	29.67	HMDB08137	C44 H83 N O8 P
	Phospholipases Triacylglycerol biosynthesis	α 2-lysophosphatidylcholine	LysoPC(20:4(5Z,8Z,11Z, 14Z)) Esi+19.0475	−329741.62	C04230	543.3323	19.05	HMDB10395	C28 H51 N O7 P
			LysoPC(20:4(5Z,8Z, 11Z,14Z)) Esi+18.974894	−3.0091927	C04230	543.3331	18.97	HMDB10395	C28 H51 N O7 P
			LysoPC(18:2(9Z,12Z)) Esi+18.912987	−2.873876	C04230	519.3315	18.91	HMDB10386	C26 H51 N O7 P
			LysoPC(18:1(11Z)) Esi+19.676394	−2.8344128	C04230	521.347	19.68	HMDB10385	C26 H53 N O7 P
			LysoPC(18:1(11Z)) Esi+19.697508	−349893.75	C04230	521.3491	19.70	HMDB10385	C26 H53 N O7 P
			LysoPC(15:0) Esi+20.451206	−35.870865	C04230	481.3185	20.45	HMDB10381	C23 H49 N O7 P
			LysoPC(22:6(4Z,7Z,10Z, 13Z,16Z ,19Z)) Esi+19.039494	−43190.832	C04230	567.3321	19.04	HMDB10404	C30 H51 N O7 P
			LysoPC(22:4(7Z,10Z, 13Z,16Z))	−7.058939	C04230	571.3657	19.95	HMDB10401	C30 H55 N O7 P
	Glycine degradation (creatine biosynthesis) Arginine degradation I (arginase pathway) Urea cycle Proline biosynthesis II (from arginine) Arginine biosynthesis IV Arginine degradation VI (arginase 2 pathway) tRNA charging Citrulline-nitric oxide cycle	L-arginine	Amino acid(Ar g-) Esi+1.134	−107148.875	C02385	174.1113	1.13		C6 H14 N4 O2
	Taurine biosynthesis	Taurine	Taurine	−5.44779	C00245	125.0148	1.35	HMDB00251	C2 H7 N O3 S
	L-dopa degradation	3-methoxy-4-hydroxyphenyllactate	Vanillactic acid	−2.3641844		212.0681	1.21	HMDB00913	C10 H12 O5
	Phosphatidylethanolamine biosynthesis II Phosphatidylethanolamine biosynthesis III	an L-1-phosphatidyl-ethanolamine	PE(15:0/18:0)	−9.105209	C00350	705.529	29.71	HMDB08892	C38 H76 N O8 P
	CMP-N-acetylneuraminate biosynthesis I (eukaryotes)	CMP-N-acetyl-Beta-neuraminate	CMP-N-acetylneuraminic acid	−183327.55	C00128	614.1504	1.49	HMDB01176	C20 H31 N4 O16 P
	Ceramide biosynthesis	Sphinganine	Sphinganine	−2.3792727	C00836	283.2875	24.44	HMDB00269	C18 H39 N O2
UP -FC ([Pext] vs. [BASAL])	Phospholipases Triacylglycerol Biosynthesis Chloline Biosynthesis III Phosphatidylcholine biosynthesis I Sphingomyelin metabolism	α phosphatidylcholine	PC(20:2(11Z,14Z)/14:0)	80.93312	C00157	757.5639	29.69	HMDB08328	C42 H81 N O8 P
			PC(20:2(11Z,14Z) /20:1(11Z))	3.9399364	C00157	821.619	29.68	HMDB08341	C48 H91 N O8 P
			PC(22:4(7Z,10Z, 13Z,16Z)/15:0)	2.9825685	C00157	777.566	29.66	HMDB08625	C45 H83 N O8 P
			PC(20:3(8Z,11Z, 14Z)/18:2(9Z,12Z)) Esi+29.711	286393.6	C00157	807.5796	29.71	HMDB08402	C46 H83 N O8 P
			PC(20:1(11Z)/18:3 (9Z,12Z,15Z))	711489.56	C00157	809.595	29.69	HMDB08305	C46 H85 N O8 P
			PC(20:1(11Z)/18:3 (9Z,12Z,15Z)) Esi+24.885	179392.34	C00157	809.5954	24.88	HMDB08305	C46 H85 N O8 P
			PC(24:0/14:0)	497.9062	C00157	799.6408	29.70	HMDB08755	C46 H93 N O8 P
	Phospholipases Triacylglycerol Biosynthesis	α 2-lysophosphatidylcholine	LysoPC(16:0) Esi+19.297081	3.063488	C04230	495.3322	19.30	HMDB10382	C24 H51 N O7 P
			LysoPC(18:2(9Z,12Z)) Esi+18.912987	2.4683728	C04230	519.3315	18.91	HMDB10386	C26 H51 N O7 P
			LysoPC(18:1(11Z)) Esi+19.676394	2.1363783	C04230	521.347	19.68	HMDB10385	C26 H53 N O7 P
			LysoPC(22:5(4Z,7Z, 10Z,13Z,16Z))	2.1225455	C04230	569.3519	19.33	HMDB10402	C30 H53 N O7 P
	Stearate biosynthesis I (animals)	Palmitic acid	Palmitic acid	4.0771503	C00249	238.2284	23.59	HMDB00220	C16 H32 O2
	NADH repair	(S)-NADHX	(6S)-6-Hydroxy-1,4,5,6-tetrahydronicotinamide-adenine dinucleotide	50127.035	C04856	683.1358	1.32		C21 H31 N7 O15 P2
	Noradrenaline and adrenaline degradation	Metanephrine	Metanephrine	3.8584461	C05588	179.0941	7.15	HMDB04063	C10 H15 N O3
	Ceramide biosynthesis	Sphinganine	Sphinganine	11.79135	C00836	283.2875	24.44	HMDB00269	C18 H39 N O2
DOWN -FC ([Pext] vs. [BASAL])	Triacylglycerol biosynthesis Phospholipases Sphingomyelin metabolism Phosphatidylcholine biosynthesis I Choline biosynthesis III	α phosphatidylcholine	PC(20:3(8Z,11Z,14Z)/18:2 (9Z,12Z))	−1091185.2	C00157	785.5968	29.69	HMDB08402	C46 H83 N O8 P
			PC(18:0/18:1(11Z))	−522777.03	C00157	769.5844	29.68	HMDB08037	C44 H87 N O8 P
			PC(18:2(9Z,12Z)/ 18:1(9Z))	−870785.8	C00157	783.5799	29.67	HMDB08137	C44 H83 N O8 P
			PC(18:0/22:5(7Z, 10Z,13Z,16Z,19Z))	−21667.828	C00157	817.6028	29.69	HMDB08056	C48 H87 N O8 P
			PC(22:0/14:0)	−2.1418252	C00157	771.6125	29.68	HMDB08525	C44 H89 N O8 P
			PC(24:0/18:2 (9Z,12Z))	−56819.664	C00157	851.6709	29.67	HMDB08763	C50 H97 N O8 P
	Triacylglycerol biosynthesis Phospholipases	α 2-lysophosphatidylcholine	LysoPC(18:1(11Z)) Esi+19.697508	−349893.75	C04230	521.3491	19.70	HMDB10385	C26 H53 N O7 P
			LysoPC(20:4(5Z,8Z, 11Z,14Z)) Esi+19.0475	−329741.62	C04230	543.3323	19.05	HMDB10395	C28 H51 N O7 P
			LysoPC(15:0) Esi+20.451206	−8.41981	C04230	481.3185	20.45	HMDB10381	C23 H49 N O7 P
			LysoPC(22:6(4Z,7Z,10Z, 13Z,16Z,19Z)) Esi+19.039494	−43190.832	C04230	567.3321	19.04	HMDB10404	C30 H51 N O7 P
DOWN -FC ([Pext] vs. [BASAL])	Phosphatidylethanolamine biosynthesis II Phosphatidylethanolamine biosynthesis III	an L-1-phosphatidyl-ethanolamine	PE(15:0/18:0)	−38.950047	C00350	705.529	29.71	HMDB08892	C38 H76 N O8 P
	Taurine biosynthesis	Taurine	Taurine	−549665.1	C00245	125.0148	1.35	HMDB00251	C2 H7 N O3 S
DOWN -FC ([Pext] vs. [BASAL])	Arginine biosynthesis IV Arginine degradation VI (arginase 2 pathway) Urea Cycle Proline biosynthesis II (from arginine) Glycine degradation (creatine biosynthesis) Arginine degradation I (arginase pathway) tRNA charging Citrulline-nitric oxide cycle	L-arginine	Amino acid(Arg-) Esi+1.134	−107148.875	C02385	174.1113	1.13		C6 H14 N4 O2
	Ceramide biosynthesis	Sphinganine	Sphinganine Esi+18.641169	−8082.4424	C00836	301.2969	18.64	HMDB00269	C18 H39 N O2
	CMP-N-acetylneuraminate biosynthesis I (eukaryotes)	CMP-N-acetyl-Beta-neuraminate	CMP-N-acetylneuraminic acid	−183327.55	C00128	614.1504	1.49	HMDB01176	C20 H31 N4 O16 P
	NAD salvage pathway II NAD salvage pathway III	1-(Beta-D ribofuranosyl) nicotinamide	Nicotinamide riboside	−81.416725	C03150	236.0834	2.23	HMDB00855	C11 H15 N2 O5
	L-dopa degradation	3-methoxy-4-hydroxyphenyllactate	Vanillactic acid	−19271.553		212.0681	1.21	HMDB00913	C10 H12 O5
UP FC (LPS vs. BASAL)	Phospholipases Triacylglycerol biosynthesis Sphingomyelin metabolism Phosphatidylcholine biosynthesis I	α phosphatidylcholine	PC(18:0/22:5(7Z,10Z,13Z, 16Z,19Z))	6.494802	C00157	817.6028	29.69	HMDB08056	C48 H87 N O8 P
			PC(20:1(11Z)/18:3 (9Z,12Z,15Z))	685521.7	C00157	809.595	29.69	HMDB08305	C46 H85 N O8 P
			PC(15:0/20:3(8Z, 11Z,14Z))	271098.25	C00157	729.5682	29.65	HMDB07948	C43 H81 N O8 P
			PC(18:2(9Z, 12Z)/20:0)	477799.22	C00157	773.6164	29.69	HMDB08143	C46 H89 N O8 P
			PC(18:2(9Z, 12Z)/20:0) Esi+29.688164	545871.8	C00157	773.6181	29.69	HMDB08143	C46 H89 N O8 P
			PC(20:4(8Z,11Z, 14Z,17Z)/20:2(11Z,14Z))	135875.12	C00157	833.5961	29.68	HMDB08473	C48 H85 N O8 P
	Phospholipases Triacylglycerol biosynthesis	α 2-lysophosphatidylcholine	LysoPC(22:5(4Z,7Z,10Z, 13Z,16Z))	8.51512	C04230	569.3519	19.33	HMDB10402	C30 H53 N O7 P
	Phospholipases Sphingomyelin metabolism Phosphatidylcholine biosynthesis I	Phosphocholine	Phosphocholine Esi+1.349	76218.5	C00588	183.0759	1.35	HMDB01565	C5 H15 N O4 P
	Stearate biosynthesis I (animals)	Palmitic acid	Palmitic acid	2.725489	C00249	238.2284	23.59	HMDB00220	C16 H32 O2
DOWN FC (LPS vs. BASAL)	Phospholipases Phosphatidylcholine biosynthesis I Sphingomyelin metabolism Choline biosynthesis III Triacylglycerol biosynthesis	α phosphatidylcholine	PC(20:1(11Z)/16:0)	−3.3726473	C00157	787.6126	29.68	HMDB08298	C44 H87 N O8 P
			PC(18:2(9Z,12Z)/ 18:1(9Z))	−870785.8	C00157	783.5799	29.67	HMDB08137	C44 H83 N O8 P
			PC(20:2(11Z, 14Z)/14:0)	−19412.555	C00157	757.5639	29.69	HMDB08328	C42 H81 N O8 P
			PC(18:0/22:5(7Z,10Z, 13Z,16Z,19Z)) Esi+29.676998	−335793.75	C00157	795.6038	29.68	HMDB08056	C48 H87 N O8 P
			PC(20:0/18:3(9Z, 12Z,15Z))	−117313.516	C00157	811.605	29.68	HMDB08272	C46 H87 N O8 P
			PC(22:2(13Z,16Z)/ 20:1(11Z))	−112728.266	C00157	827.6702	29.68	HMDB08603	C50 H95 N O8 P
			PC(18:2(9Z,12Z)/22:0)	−121347.46	C00157	823.6375	29.69	HMDB08150	C48 H93 N O8 P
			PC(22:4(7Z,10Z, 13Z,16Z)/15:0)	−27570.09	C00157	777.566	29.66	HMDB08625	C45 H83 N O8 P
			PC(24:0/18:2(9Z,12Z))	−56819.664	C00157	851.6709	29.67	HMDB08763	C50 H97 N O8 P
	Phospholipases Triacylglycerol biosynthesis	α 2-lysophosphatidylcholine	LysoPC(20:4(5Z,8Z, 11Z,14Z))	−286604.6	C04230	543.3326	19.13	HMDB10395	C28 H51 N O7 P
	Phospholipases Phosphatidylcholine biosynthesis I Sphingomyelin metabolism Choline biosynthesis III	Phosphocholine	Phosphocholine	−77069.34	C00588	183.0663	1.35	HMDB01565	C5 H15 N O4 P
	Arginine biosynthesis IV Arginine degradation I (arginase pathway) Arginine degradation VI (arginase 2 pathway) Glycine degradation (creatine biosynthesis) Proline biosynthesis II (from arginine) Citrulline-nitric oxide cycle	L-arginine	Amino acid(Arg-) Esi+1.296263	−152038.48	C02385	174.1113	1.30		C6 H14 N4 O2
			Amino acid(Arg-) Esi+1.134	−107148.875	C02385	174.1113	1.13		C6 H14 N4 O2
	Noradrenaline and Adrenaline degradation	Metanephrine	Metanephrine	−11.955253	C05588	179.0941	7.15	HMDB04063	C10 H15 N O3
	Phosphatidylethanolamine biosynthesis II Phosphatidylethanolamine biosynthesis III	an L-1-phosphatidyl-ethanolamine	PE(15:0/18:0)	−74.26719	C00350	705.529	29.71	HMDB08892	C38 H76 N O8 P
	Ceramide biosynthesis Urea Cycle	Sphinganine	Sphinganine	−3.8664813	C00836	283.2875	24.44	HMDB00269	C18 H39 N O2
			Sphinganine Esi+18.641169	−2.807113	C00836	301.2969	18.64	HMDB00269	C18 H39 N O2
	CMP-N-acetylneuraminate biosynthesis I (eukaryotes)	CMP-N-acetyl-Beta-neuraminate	CMP-N-acetylneuraminic acid	−183327.55	C00128	614.1504	1.49	HMDB01176	C20 H31 N4 O16 P

Entities or compound classes that have been found were: α-phosphatidylcholine (PC), α-2-lysophosphatidylcholine (LysoPC), L-arginine (Arg(-)), metanephrine, (S)-NADHX, palmitic acid, sphinganine, phosphocholine, taurine, 3-methoxy-4-hydroxyphenyllactate, an-L-1-phosphatidyl-ethanolamine (PE), CMP-N-acetyl-β-neuraminate (CMP-Neu5Ac) and 1-(β-D ribofuranosyl)nicotinamide. Among metabolic pathways, in which these entities are involved, were found representatives of lipid metabolism such as phospholipases metabolism (PC, LysoPC and phosphocholine) and biosynthetic pathways of triacylglycerol (PC and LysoPC), sphingomyelin, choline, phosphatidylcholine (PC and phosphocholine), phosphatidylethanolamine (PE), ceramide (sphinganine), and stearate (palmitic acid). Amino acid metabolism pathways such as arginine metabolism, urea and citrulline-nitric oxide cycle, proline biosynthesis, glycine degradation, tRNA charging (L-Arg(-)) and taurine biosynthesis (taurine). Representatives of catecholamine metabolism such as L-dopamine (3-methoxy-4-hydroxyphenyllactate), noradrenaline and adrenaline (metanephrine) degradation. In addition, entities involved in NAD salvage (1-(β-D ribofuranosyl) nicotinamide), NADH repair ((S)-NADHX) and CMP-N-acetylneuraminate biosynthesis (CMP-Neu5Ac) pathways were also found related with this study.

#### Common metabolomics response to the different microbiota-derived molecules

Results interpretation was quite complex as some of the entities were found both over and underproduced after incubation with either LPS, peptide HM14 or Pext, such as α-phosphatidylcholine, 2-lysophosphatidylcholine, L-arginine or sphinganine. For instance, in the case of sphinganine entity, two compounds were putatively identified, sphinganine Esi+18.641169 (302.2969 m/z; 18.64 min) and sphinganine (284.2875 m/z; 24.44 min). As it has been reported by (Mi et al., 2016), fragment at 284.3 m/z can result from the fragmentation of both protonated adducts of sphinganine, as a result of the dehydration of their protonated molecular ion, and sphinganine-1-phosphate, through loss of phosphoric acid from the molecular structure, in positive ion mode. Since sphinganine-1-phosphate elutes later than sphinganine, this suggest that sphinganine (284.2875 m/z; 24.440 min) could come from the phosphorylated form and therefore, lead to the identification of two different compounds with the same value of m/z and different retention time that appears both over and under-expressed within the same condition.

Another example is phosphocholine, head group of one class of phospholipids, with a predominant fragment at 184 m/z, which could be produced by the fragmentation, in positive ion mode, of protonated adducts of phosphatidylcholines and sphingomyelins (Jackson et al., 2005). This could explain the reason why two molecular ions of phosphocholine, phosphocholine (183.0663 m/z; 1.35 min) and phosphocholine Esi+1.349 (183.0759 m/z; 1.35 min), were found both in over and underproduction in LPS condition, being the response to HM14 an overproduction of the compound phosphocholine in contrast to the response observed against LPS.

L-arginine was also found both up- and down-regulated by HM14, through the ionic forms of Amino acid(Arg-) Esi+1.134 (174.1113 m/z; 1.13 min) and Amino acid(Arg-) Esi+1.296263 (174.1113 m/z; 1.30 min). After a visual inspection of the chromatogram, due to the peak form, it could be possible that both ionic forms belonged to the same molecular structure. Moreover, although the software identified the entity name as L-arginine, D-arginine could also be present and this fact could explain the peak form as well (Han et al., 2018). Thus, further quantitative analysis by MS/MS is needed to identify this compound and to know the magnitude of the change and therefore, pathways involved (such as arginine biosynthesis/degradation) will not be discussed at this time. However, it is noteworthy that Pext only down-regulated the concentration of this amino acid, suggesting that Pext may be interacting through different mechanisms with the immune cell populations. Therefore, as the implication of different ionic forms corresponding to the same molecules requires a more exhaustive understanding of the metabolic response of the different immune cells to the molecules involved in this work, the metabolic pathways corresponding to these compounds will not be evaluated at this time. Further experimentation with cellular subsets may identify the immune populations, in which a given pathway is up or down-regulated. As well as performing a targeted MS/MS analysis, it will be possible to elucidate fragmentation patterns that allow the identification of each molecular structure.

Among the entities that showed down-regulation patterns as affected by HM14, Pext and LPS we identified CMP-Neu5Ac and L-1-phosphatidyl-ethanolamine. CMP-Neu5Ac is the activated form of 5-N-Acetylneuraminic acid (Neu5Ac), the major sialic acid found in animal cell surfaces (Li and Chen, 2012). Alterations on cell surface glycoconjugates by removal of terminal sialic acids residues by sialidases influence cellular activity. (Nan et al., 2007) showed an association of desialylation of specific glycoconjugates on the cell surface of activated human T lymphocytes, and suggested a correlation between sialidase inhibition and decreases in the production of IFN-γ (Stamatos et al., 2004). Regarding PE, it is the second most abundant lipid on cellular membranes and plays an important role in membrane fusion, cell cycle, autophagy and apoptosis (Pavlovic and Bakovic, 2013). It can be *de novo* synthesized from ethanolamine, which plays a significant role in modulating intestinal inflammation and in the proliferation and differentiation of intestinal cells (Zhou et al., 2017). (Martins et al., 2016) also reported decreased PE concentrations in THP-1 cells exposed to methyl salicylate, a respiratory irritant chemical. Decreases in these two metabolites may represent common response pathways to conserved microbial-associated molecules.

**Figure 6:**
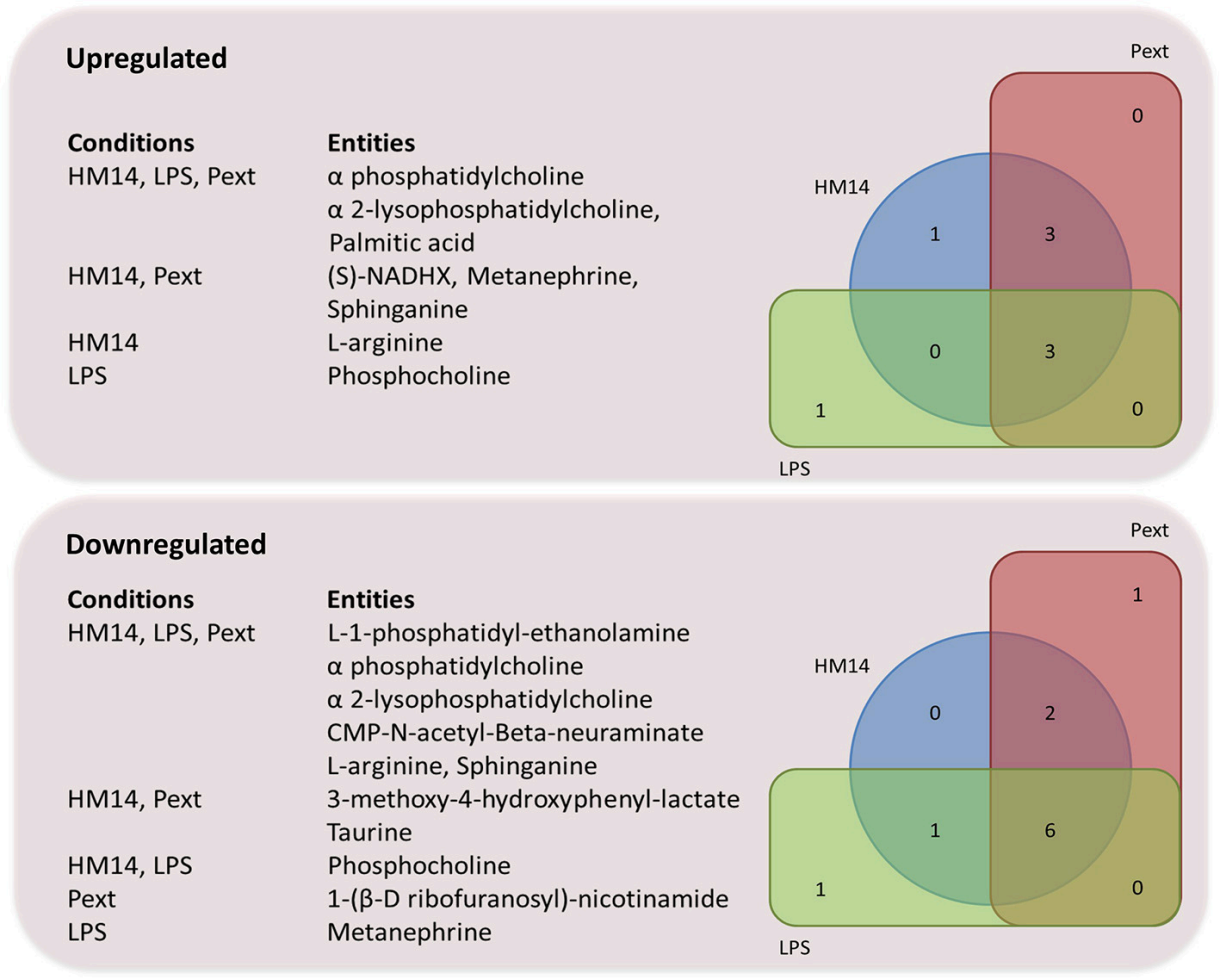
Venn diagram representing over-and under-produced entities, referred to basal conditions, after MPP pathway analysis.

A deeper analysis into what metabolites increased/decreased their intracellular concentrations following challenges with LPS, HM14 peptide or Pext fraction, revealed interesting patterns. Figure 6 represents Venn diagrams of over- and under-produced metabolites, always referred to basal conditions, in the different experimental settings as well as their intersections. It is noteworthy the amount of metabolites downregulated by the three bacterial molecules: L-1-phosphatidyl-ethanolamine, phosphatidylcholine, 2-lysophosphatidylcholine, CMP-N-acetyl-β-neuraminate, L-arginine and sphinganine. This suggests some kind of central response to commensal bacteria components, regardless their biochemical nature (proteic or lipoglucidic) or if they are originated from a potential pathogen (*E. coli*) or a potential probiotic (*L. acidophilus*). The boundary between both bacterial features (probiotic vs. pathogen) is somehow diffuse and strain-dependent, and we can find either *E. coli* probiotic strains such as strain Nissle 1917 (Henker et al., 2008), or *Lactobacillus* sp. strains (Martinez et al., 2014) contributing to infections.

#### Specific HM14 and pext immunological effects

**Figure 7:**
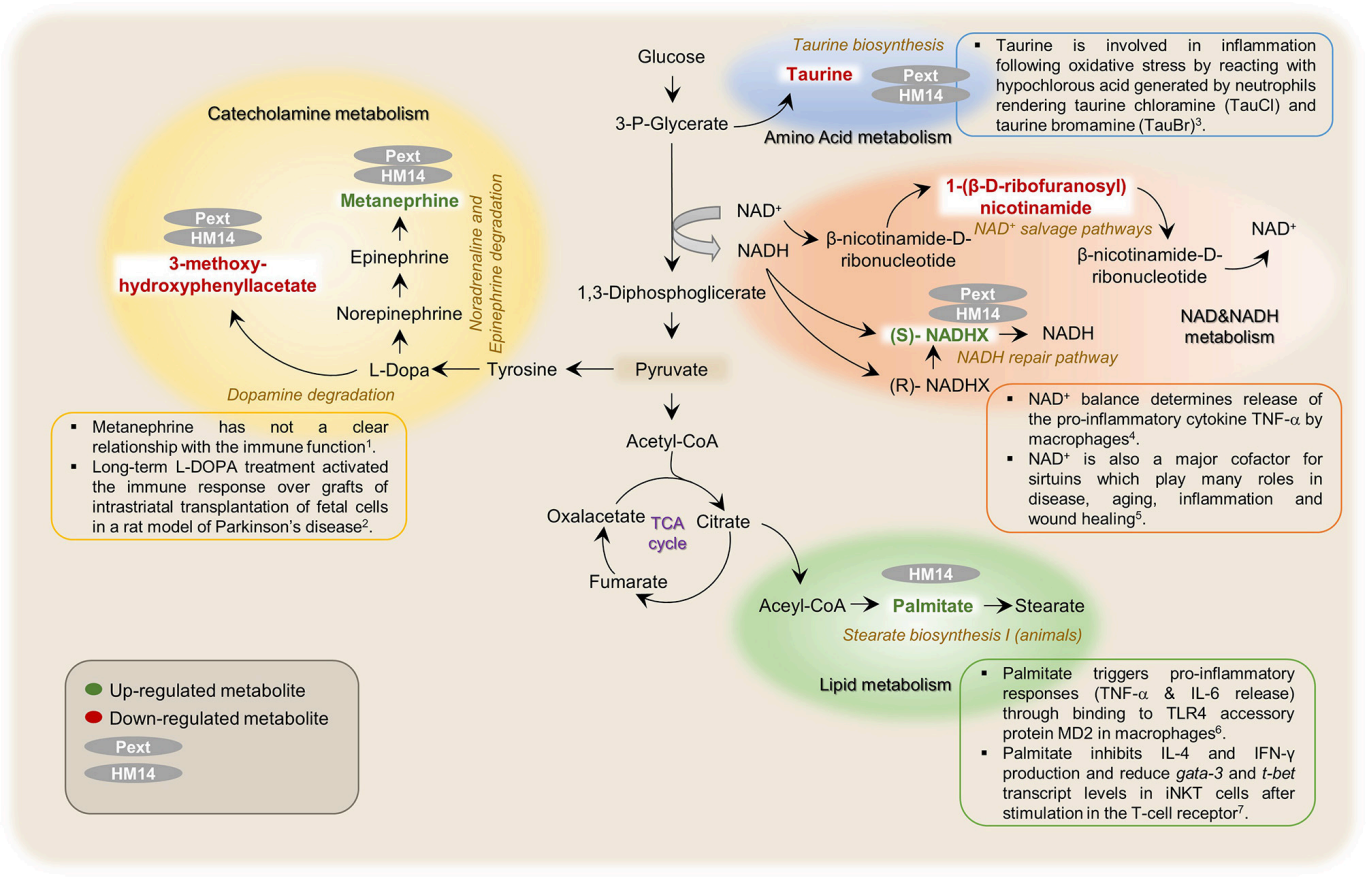
Schematic representation of metabolic pathways that were found related with the possible immunomodulatory effects induced by the studied proteinaceous molecules (HM14 and Pext) over PBMCs. ^1^(Breger et al., 2017), ^2^(Marcinkiewicz and Kontny, 2014), ^3^((Al-Shabany et al., 2016), ^4^(Qiang et al., 2017), ^5^(Wang et al., 2017), and ^6^(Ko et al., 2017).

It is noteworthy that both intestinal bacteria derived molecules, HM14 and Pext, induced metabolic responses that somehow diverged from *E. coli* LPS. A schematic representation of the possible metabolic pathways in which these molecules may be involved is shown in Figure 7. Among the compounds upregulated specifically by the presence of peptide HM14 we found palmitic acid, which participates in the stearate biosynthesis I pathway. Palmitic acid is by far the most abundant saturated fatty acid in living beings, but it is well-known in our society by being the major component of palm oil, being present in a wide array of food products. This long chain fatty acid interacts with toll-like receptor 4 (TLR4) expressing cells, such as dendritic cells (Nicholas et al., 2017). Palmitic acid also binds to the TLR4 accessory protein MD2 and triggers pro-inflammatory responses, both *in vivo* (mouse model) and *in vitro*, being the mechanisms by which circulating palmitic acid induces myocardial injury (Wang et al., 2017). Indeed md2 -/- mice are protected against the pro-inflammatory effects under a high-fat diet. A recent report has indicated that either a palmitic acid-rich diet or injection supressed arthritis in a mouse model by modulating directly the function of invariant natural killer T (iNKT) cells, being able to decrease antibody-induced joint inflammation. This was elicited through an inhibition of the pro-inflammatory mediators IL-4 and IFN-γ and a reduction in the level of the transcription of the *gata-3* and *t-bet* genes, after stimulation in the T-cell receptor (Ko et al., 2017).

HM14 and Pext increased the intracellular concentrations of (S)-NADHX, an enantiomer of the NADH hydrate formed after glyceraldehyde 3-phosphate dehydrogenase action, which is bioconverted back to NADH by the action of an ATP-dependent NAD(P)H dehydratase. As a whole, these reactions form part of the so-called NADH repair pathway. NADH is the reduced form of the coenzyme nicotinamide adenine dinucleotide, a well-known electron carrier in redox reactions. NAD^+^ balance determines release of the pro-inflammatory cytokine TNF-α by macrophages in response to LPS, which signals through TLR4 (Al-Shabany et al., 2016). Interestingly, LPS did not affect the NADHX levels in our experimental setting. NAD^+^ is also a major cofactor for sirtuins, which are a family of 7 proteins with deacetylase and ADP ribosyltransferase activities. These proteins play many roles in disease, aging, inflammation and wound healing (Qiang et al., 2017). Finally, NAD^+^ has also been identified as an extracellular signaling molecule, being for instance released by neurons in the large intestine with inhibitory activity over the smooth muscle surrounding the intestinal mucosa (Mutafova-Yambolieva et al., 2007; Hwang et al., 2011).

Both HM14 and Pext increased also the concentration of metanephrine, a metabolite from the noradrenaline/adrenaline degradation. This molecule has not a clear relationship with the immune function, but higher plasma levels have been correlated to a high risk of prediabetes, and it has been also proposed as a biomarker for pheochromocytoma, a type of neuroendocrine tumor affecting the adrenal glands (Lenders et al., 2002; Wang et al., 2015). Interestingly, the effect of LPS was inverse as this pro-inflammatory compound decreased the levels of metanephrine. Therefore, unless requiring more experimental evidence, this might be interpreted as an anti-inflammatory effect as being opposite to what is observed for LPS.

Regarding the metabolites downregulated by the incubation of HM14 and Pext over PBMCs, our analysis identified 3-methoxy-4-hydroxyphenyl-lactate involved in L-DOPA degradation and the amino acid taurine. L-DOPA is the metabolic precursor of dopamine and it is among the most effective treatments for Parkinson's disease. In a recent paper, it has been observed that long-term L-DOPA treatment activated the immune response over both allogeneic and xenogeneic grafts of intrastriatal transplantation of fetal cells, even in the presence of the immunesupressor cefalosporine (Breger et al., 2017). In the same way, it has been suggested a role for taurine in inflammation following oxidative stress by reacting with hypochlorous acid generated by neutrophils rendering taurine chloramine (TauCl) and taurine bromamine (TauBr) (Marcinkiewicz and Kontny, 2014). Therefore, an anti-inflammatory role can be proposed to both molecules that will deserve further experimentation.

Finally, Pext decreased the concentration of 1-(β-D ribofuranosyl)-nicotinamide in PBMCs, a metabolite involved in the NAD salvage pathway II and III. As pointed out before, NAD^+^ is a central coenzyme for energy metabolism, cell growth and many others housekeeping pathways. Inhibition of this pathway and therefore the NAD^+^ levels combined with many cancer treatments returned often higher cytotoxicity over cancer cells (Kennedy et al., 2016).

## Conclusions

In conclusion, an untargeted metabolic fingerprinting was performed in order to evaluate intracellular changes in PBMCs, in the presence of proteinaceous molecules derived from gut bacteria and a pro-inflammatory endotoxin, by using LC-QTOF-MS. This, reinforces the importance of metabolomics approaches to better understanding immune cell response. Results suggest that HM14 and Pext, a peptide derived from an extracellular protein of *B. longum* and the extracellular protein fraction of *L. acidophilus*, induced metabolic responses in PBMCs that diverged from those observed for the pro-inflammatory *E. coli* LPS. NADH arrest, NAD^+^ reduction, as well as increases in palmitic acid and methanephrin, would support an anti-inflammatory molecular mechanism of action of these two molecules, although this will require further studies using human translational models for inflammation: skin patch.

## Author contributions

BS and EM-C conceived the experiments. RA-A and MM-G designed the immunological study. NC-P, CH-C, and MM-G performed biological experiments. NC-P, EM-C, and JS-G performed metabolomics analysis. NC-P, EM-C, BS, and JS-G wrote the manuscript. The manuscript was reviewed by all the authors.

### Conflict of interest statement

The authors declare that the research was conducted in the absence of any commercial or financial relationships that could be construed as a potential conflict of interest.
